# Culture Modulates Eye-Movements to Visual Novelty

**DOI:** 10.1371/journal.pone.0008238

**Published:** 2009-12-16

**Authors:** Joshua O. Goh, Jiat Chow Tan, Denise C. Park

**Affiliations:** 1 Beckman Institute, University of Illinois, Urbana-Champaign, Illinois, United States of America; 2 Cognitive Neuroscience Laboratory, Duke-NUS Graduate Medical School, Singapore, Singapore; 3 Center for Brain Health, University of Texas, Dallas, Texas, United States of America; National Institute of Mental Health, United States of America

## Abstract

**Background:**

When viewing complex scenes, East Asians attend more to contexts whereas Westerners attend more to objects, reflecting cultural differences in holistic and analytic visual processing styles respectively. This eye-tracking study investigated more specific mechanisms and the robustness of these cultural biases in visual processing when salient changes in the objects and backgrounds occur in complex pictures.

**Methodology/Principal Findings:**

Chinese Singaporean (East Asian) and Caucasian US (Western) participants passively viewed pictures containing selectively changing objects and background scenes that strongly captured participants' attention in a data-driven manner. We found that although participants from both groups responded to object changes in the pictures, there was still evidence for cultural divergence in eye-movements. The number of object fixations in the US participants was more affected by object change than in the Singapore participants. Additionally, despite the picture manipulations, US participants consistently maintained longer durations for both object and background fixations, with eye-movements that generally remained within the focal objects. In contrast, Singapore participants had shorter fixation durations with eye-movements that alternated more between objects and backgrounds.

**Conclusions/Significance:**

The results demonstrate a robust cultural bias in visual processing even when external stimuli draw attention in an opposite manner to the cultural bias. These findings also extend previous studies by revealing more specific, but consistent, effects of culture on the different aspects of visual attention as measured by fixation duration, number of fixations, and saccades between objects and backgrounds.

## Introduction

A number of studies have now demonstrated that the cultural experience of individuals has a significant effect on how visual information is processed. Western cultures emphasize independence, and individuals who come from these cultures tend to be more analytic and process visual stimuli with a focus on objects and their attributes. In contrast, East Asian cultures emphasize interdependence, which leads to more monitoring and holistic processing of contextual information [1]–[6]. For example, Kitayama et al. [7] showed that when making judgments about the length of lines drawn within a box frame, responses in East Asians were more affected by the size of the contextual box frame compared to Westerners. In another study involving change-detection with complex pictures, East Asians noticed visual changes occurring in the background more frequently than Westerners [8]. These studies, amongst many others [9], point to differences between Westerners and East Asians in the attention to different elements within a visual scene. In this present study, we evaluated the robustness and specific mechanisms of these cultural differences in visual attention as participants from East Asian and Western cultures viewed complex scenes that contained focal objects and backgrounds that were selectively changed in order to capture subjects' attention in a data-driven manner.

In a study of visual scene processing, Chua et al. [10] demonstrated that there were cultural biases in the eye-movements of American and Chinese participants as they viewed complex pictures containing central objects within a background scene. Americans spent a greater proportion of viewing time on objects relative to backgrounds compared to Chinese participants. While the number of object fixations was similar for both groups, Chinese made more background fixations than Americans. In addition, in Americans, the average duration of object fixations was longer than for background fixations, and this difference was greater than in Chinese. Interestingly, Chua et al. [10] also observed that overall durations for *both* object and background fixations were longer for Americans than for Chinese, indicating that Americans' eye-movements were characterized by longer dwell times, perhaps to extract more visual details at each focal point. These eye-movement findings were consistent with a more analytic style of processing in the Americans as Westerners and a more holistic processing style in the Chinese as East Asians.

Recent neuroimaging studies have extended these findings of cultural differences in visual processing of scenes to patterns of neural activation in ventral visual and parietal regions of the brain. When processing complex pictures of objects in scenes, object-processing brain regions in the ventral visual areas in East Asians were characterized by reduced engagement compared to Westerners [11], [12]. These highly specialized areas are involved in perceptual analysis and processing, although top-down attentional processes may also modulate brain activity in these regions [13], [14]. In addition, Hedden et al. [15] conducted an fMRI study using the same line judgment task as in Kitayama et al.'s [7] behavioral study, and reported more brain activity when individuals from each culture performed the task using their culturally non-preferred strategy. Specifically, there was increased parietal engagement (a visuo-spatial attentional region) when East Asians made absolute judgments compared to relative judgments, whereas Westerners showed increased parietal activity when making relative judgments compared to absolute judgments. The findings in the parietal cortex reflect cultural differences in the effort associated with performing a specific strategic task whereas the ventral visual findings suggest that perceptual mechanisms in the brain are analyzing somewhat different scene elements as a function of culture. Taken together, these data suggest that culture operates as a top-down mechanism that guides and interacts with basic neuro-perceptual processes.

Given these cultural differences when Westerners and East Asians view typical scenes, an important question is how the contents of a scene, and changes in it, might interact with top-down cultural biases to process scene information. That is, are the cultural differences observed during scene viewing in Chua et al. [10] always present regardless of the scene composition and viewing conditions? And what are the specific cognitive mechanisms involved? In another eye-tracking study by Rayner et al. [16], it was found that when scenes contained several objects of interest, cultural differences in the number of fixations were minimal between Westerners and East Asians. When examining eye-movements for a subset of their scene stimuli that consisted of singular central objects, their data more resembled Chua et al.'s [10] findings, with East Asian participants making more background fixations than Western participants. Such findings suggest that cultural differences during scene viewing may be susceptible to the composition and salient changes in the visual stimuli.

It is also well known that onsets of novel salient visual stimuli capture attention in a data-driven manner involving cognitive processes that are independent of the individual's experience or goals, but dependent on the external stimuli [17]–[20]. Responses to these external stimuli are not easily inhibited [21], [22] and exert a strong influence on eye-movements, such as demonstrated in anti-saccade paradigms [23], [24]. Even so, in tandem with such data-driven processes, top-down processes that are dependent on the individuals' experience and goals also affect attention. Visual orienting cues, task demands and past experiences with repeated stimuli can modulate the effects of data-driven distractor stimuli on behavioral responses [20], [25]–[28], as well as eye-movements [23], [29]. This suggests that top-down processes can at times over-ride or even enhance the data-driven mechanisms involved in visual attention. We propose that cultural biases, for interpreting the same information in different ways, can be construed as a case of top-down processes acting on attention towards data-driven visual events in the environment. These biases are acquired through experience within cultural backgrounds that differentially encourage or discourage particular modes of thinking and, ultimately, cognition [3]–[5], [9].

In the present study, we used eye-tracking to investigate the relationship between top-down cultural biases in scene analysis and data-driven information present in the perceptual environment, implementing a picture repetition paradigm that was previously used to isolate cultural differences in ventral visual brain activity in a functional imaging study by Goh et al. [11]. In that study, young and old Chinese Singaporeans (East Asians) and Caucasian Americans (Westerners) were presented with complex pictures consisting of an object placed against a background scene. The objects and backgrounds were selectively repeated. With each repetition, the brain adapted or showed less activity in either the object or background processing areas, depending on which element was manipulated. Older Caucasian Americans showed more selective neural response to objects compared to older Chinese Singaporeans, consistent with more object-focused processing in the older Westerners. In the present study we used this paradigm with eye tracking to investigate the effects on visual attention in Western and East Asian participants when there are conflicts between data-driven stimulus changes that should direct eye movements towards certain scene elements, and top-down cultural processes, that also bias eye movements.

Thus, the study allowed us to address how robust cultural biases are on visual attention in the face of salient changes in the individual's visual environment. Specifically, we were interested in evaluating whether, in the presence of salient novel stimuli, top-down cultural biases to process object or background components are maintained preferentially or whether they are over-ridden by data-driven changes in the stimuli. Due to the focus on objects, and the fact that the attentional system is geared toward detecting visual novelty, an onset of object change or novelty in a complex scene should capture attention in Westerners, as one would expect in any event of visual onset [23], [29]. We expected that East Asians should show a similar attention to visual novelty. However, due to a parallel focus on holistic processing of the total scene, this effect may be dampened, resulting in a more equivalent response to object and background novelty, since any change in the visual scene would disrupt the holistic representation of the scene. If so, this finding would suggest that there are culturally different mechanisms in the responses to visual novelty that are relatively robust regardless of the visual stimuli. Given the consistency of findings on cultural effects on cognition in the behavioral, eye-tracking, and neuroimaging literature, we hypothesized that top-down cultural biases of viewing objects and backgrounds would still be expressed in the face of changing stimuli. Yet another possible outcome would be that the data-driven scene changes would override top-down cultural biases such that when picture elements are changed (either the object or background), both Westerners and East Asians' attention would be drawn towards the novelty rather than repeated elements, and cultural differences are eliminated. Such a finding would indicate that cultural biases for visual attention to objects and contexts are over-ridden by attention to visual novelty, which takes priority at least in terms of eye-movements. The extent to which cultural effects are preserved in the presence of changes in stimulus variables would have implications on how fundamental these effects are on visual processing.

With regards to projecting these cultural differences onto eye-movement variables, previous studies have quantified eye-movements during scene viewing using the number of object and background fixations and mean fixation durations. We reasoned that these measures of eye-movements reveal different facets of visual attention [29] that may be modulated differently by culture. The number of fixations made to objects and backgrounds provides information about where fixations tend to be targeted on, revealing what is visually important in the scene to direct foveal vision on for further more detailed processing. Once a fixation is made, fixation durations indicate how long higher-level focus is maintained on the same region, allowing for the extraction of greater visual details. We expected that due to a focus on objects and their attributes that is characteristic of analytic processing, Westerners would tend to fixate on the most important or salient item in the stimuli – the object, and show longer dwell times per fixation in order to extract featural details. When objects are selectively changed, Westerners should modulate eye-movements in response to this object novelty, but remain relatively unaffected by changes that occur in the background. In contrast, while objects are also important to East Asians, the context has more elevated status as well. Thus, East Asians would show more widespread points of visual focus, with shorter fixation durations, de-prioritizing featural details as they have to attend from point to point within a limited amount of time. We expected that while object change would also have an effect on East Asians' eye-movements, this effect would be less than that observed for Westerners based on the reasons described above.

## Methods

### Ethics Statement

All subjects gave written informed consent approved by the Internal Review Boards (IRB) at the University of Illinois at Urbana-Champaign and Singapore General Hospital.

### Participants

There were a total of 31 participants in this study: 16 Western volunteers from the US and 15 East Asian volunteers from Singapore. Volunteers in the US sample were all Caucasian American students recruited from the University of Illinois at Urbana-Champaign, and consisted of 7 males and 9 females, with a mean age of 21.4 years, and range: 19 to 29 yrs. Although the only group excluded from the study in the US sample was individuals of Asian descent, it was a chance occurrence that no African Americans, American Latinos or individuals of other race or ethnicity volunteered for this particular study. Volunteers in the Singapore sample were all Chinese Singaporeans and consisted of 9 male and 7 female students recruited from the local universities in Singapore, with a mean age 22.1 years, ranging from 20 to 25 yrs. Singapore is a multicultural nation-state that is closely associated with other Asian countries (such as China and Japan) and predominantly subscribes to East Asian values (http://www.geert-hofstede.com). There are three main racial groups in Singapore with their preserved cultures comprised of Chinese, Malay, and Indian cultures. Because we were interested in focusing on a single cultural group that was representative of East Asians as in previous studies, all participants were ethnic Chinese born in Singapore. Subjects with counter-indications for eye-tracking measurements were excluded. Visual acuity was corrected to 20/20 where necessary. All subjects were paid USD15 per hour as remuneration for their participation. Participants were tested in their home countries.

### Stimuli and Procedure

**Figure 1:**
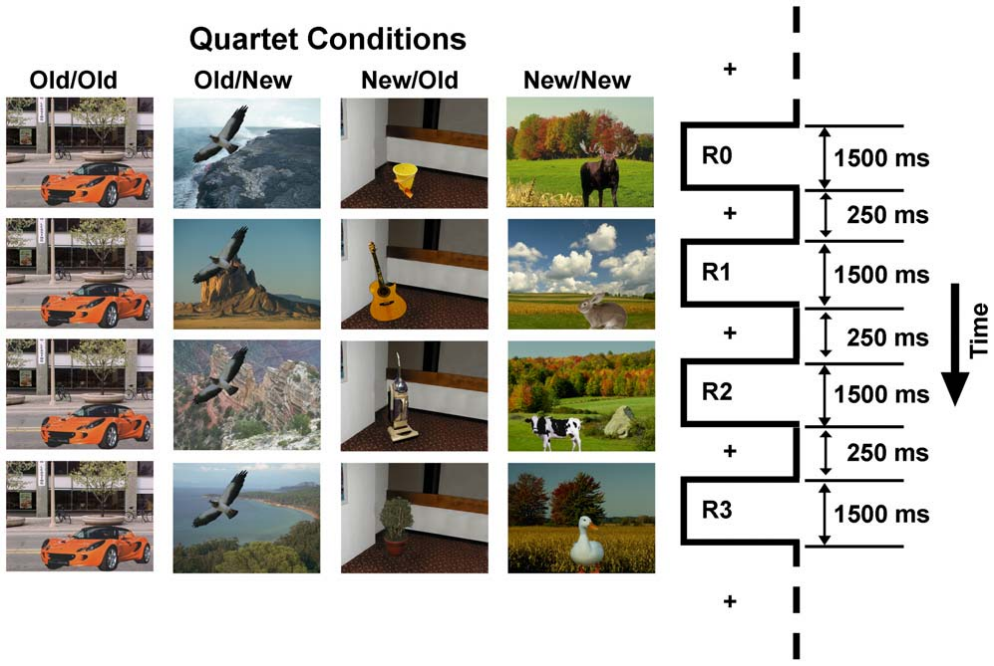
Schematic of the experimental design showing example stimuli from the four picture quartet conditions (left) and the display timings (right).

The stimuli were the same as those used in Goh et al. [11], [30] and Chee et al. [31]. Briefly, photographs of objects and backgrounds were used to compose 200 unique pictures, each consisting of an object placed within a congruent background scene (Figure 1). The pictures were grouped into quartets. There were four types of quartets, with 20 exemplars of each: 1) Old/Old: both an object and background are repeated four times without change 2) Old/New: an object is repeated four times against four changing backgrounds 3) New/Old: the object changes, but the background is repeated, and 4) New/New: both the object and background change. These four quartet conditions allowed us to examine how eye-movements are affected by data-driven changes in object/background relationships across pictures within the quartet in concert with top-down biases of cultural experience. Objects and backgrounds in each quartet were not repeated again in other quartets.

Participants were instructed to view the series of pictures and to focus on a fixation cross whenever it was presented. All subjects received the same instruction. At the beginning of each quartet, a fixation cross was presented and the eye-tracker verified fixation was present for 2000 ms before the first picture in the quartet was presented. Pictures were always displayed as a quartet, with each picture within the quartet presented for 1500 ms with an inter-picture interval of 250 ms during which a fixation cross was presented against a black screen. The visual angles subtended by the objects ranged from approximately 0.5°×1.0° to 2.5°×5.5° away from the object centers. The visual angles subtended by the backgrounds were 4.6°×6.3° away from the center fixation with subjects' eyes at a viewing distance of approximately 50 cm from the screen. Eye-movement data were recorded for each presented picture.

The same experimenter supervised data collection in both Singapore and the U.S. and great care was taken to standardize instructions and data collection procedures across the two sites. The same Tobii Eyetracker System was used at both sites with a spatial resolution of 1-degree visual angle and a sampling rate of 50 Hz (20 ms per data point). Eye-movement information obtained from the system consisted of the pixel coordinates of where participants' eyes were looking on the screen (gaze) and the timestamp of that gaze data point.

### Data Analysis

The measures of interest (fixation duration and number) assessed where in the picture, and for how long, gaze was directed. To discriminate whether each gaze was directed to the object or background, masks of the objects were created (using Matlab) that expanded the object boundaries by 15 pixels. Each gaze data point was assigned as an object or background data point based on whether their pixel coordinates fell within the resulting object masks for each picture. Fixations were then defined using an intersection of the following two criteria: 1) the clusters of gaze data points were within a 30 pixel radius of their centroid and 2) the summed temporal duration of the cluster of gaze data points was greater than 50 ms [32], [33].

Using the fixation data, we measured the average fixation duration to objects and backgrounds within each picture [29], [32]. In addition, we also measured the number of fixations made to the object and background. Together, these measures allowed us to evaluate how number of fixations and fixation viewing time was distributed between objects and backgrounds. Each of the four eye-movement measures (fixation duration to objects, fixation duration to background, number of object fixations, number of background fixations) were analyzed using three-way repeated-measures analyses of variance (ANOVA) with Culture (Singapore and US) as a between subject variable, and Quartet Condition (Old/Old, Old/New, New/Old and New/New) and Picture Repetition (R0 to R3) as within subject variables. Subsequent second-level statistical comparisons to qualify significant effects obtained from the omnibus ANOVAs and other following specific analyses are described in the corresponding results sections.

## Results

### Object Fixation Data

**Figure 2:**
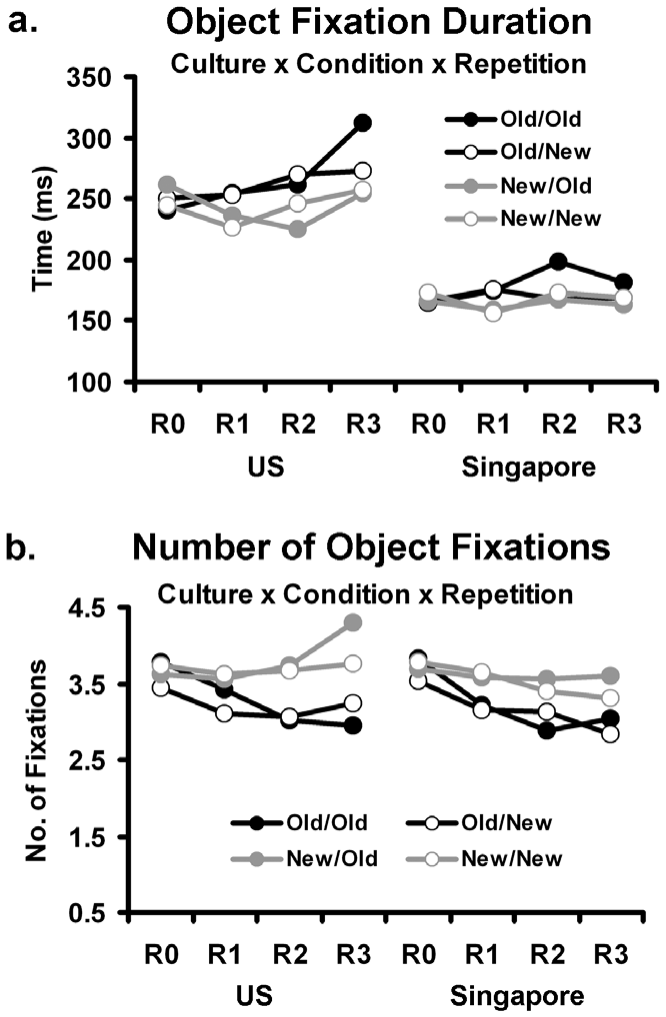
Mean object fixation data for US and Singapore participants. Mean object fixation durations (a) and number of object fixations (b) are shown across all quartet conditions (Old and New objects/backgrounds) and picture repetitions (R0 to R3).

First, the object fixation duration data will be reported, followed by the number of fixations to objects. This will be followed by similar analyses for backgrounds. For the object fixation duration analysis (Figure 2a), there was a significant main effect of culture (F(1, 29) = 8.09, p<.01, η^2^ = .22) due to the US participants having longer object fixations than the Singapore participants (254.2 ms vs. 170.3 ms). There were also main effects of condition (F(3,87) = 2.92, p<.05, η^2^ = .09) and repetition (F(3,87) = 4.23, p<.01, η^2^ = .13). The three main effects were qualified by three interactions: the two-way interaction between repetition and condition (F(9, 261) = 3.52, p<.01, η^2^ = .11), between repetition and culture (F(3, 87) = 3.13, p<.05, η^2^ = .10), and the three-way interaction of culture, condition, and repetition (F(9, 261) = 1.86, p = .06, η^2^ = .06). The two-way interactions are not discussed further since the variables in these interactions were involved in a three-way interaction. We decomposed the three-way interaction by separately examining differences in the linear effects of repetition for each condition, for each culture. The linear trend analyses we report were one-way ANOVAs of each condition and culture with linear weightings for repetition. For the US participants, there were positive linear trends over repetitions for the Old/Old (F(1, 15) = 9.39, p<.01, η^2^ = .39), and Old/New (F(1, 15) = 5.12, p<.05, η^2^ = .25) conditions but no significant linear trends for the New/Old and New/New conditions (p = .45 and .38 respectively). These results suggest that when objects were repeated during the Old/Old and Old/New conditions, fixation duration to objects in the US participants was magnified over repetitions regardless of the background changes. In contrast, in the Singapore participants, there were no significant linear effects of repetition, suggesting that duration of fixation was not modulated by data-driven changes in the study. Overall, the analysis confirmed the predicted bias of Westerners fixating longer on objects compared to East Asians when objects remained unchanged. East Asians showed less attention to objects, and did not redistribute their attention when objects or background changed.

A further analysis of object-based processing assessed the number of fixations to objects. The analysis did not yield a significant main effect of culture, but the condition (F(3, 87) = 30.65, p<.01, η^2^ = .51) and repetition (F(3, 87) = 18.64, p<.01, η^2^ = .39) main effects were significant, as were the repetition by condition (F(9, 261) = 10.31, p<.05, η^2^ = .26) and repetition by culture interactions (F(3, 87) = 6.14, p<.01, η^2^ = .18). Again, there was a three-way interaction with culture, condition and repetition (F(9, 261) = 2.32, p<.05, η^2^ = .07; Figure 2b). Based on this interaction, one-way ANOVAs of linear trends were again performed to evaluate the effect of repetition for each condition separately for each culture as detailed above. As can be seen in Figure 2b, the US group showed a linear decrease in number of fixations for Old/Old (F(1, 15) = 26.08, p<.01, η^2^ = .64) and a linear increase in number of fixations to objects for New/Old (F(1, 15) = 29.32, p<.01, η^2^ = .66) conditions. There were no significant linear trends when backgrounds were changing (Old/New and New/New). This shows that the US participants exhibited fewer fixations to objects over time if it were repeated amidst a constant background. If the object was changing, however, fixations to the objects became more frequent, as the US participants conceivably devoted more attentional resource to attend to the novel object information. In contrast, for the Singapore participants, the number of fixations to objects significantly decreased across repetitions for all conditions (Old/Old: F(1, 14) = 21.54, p<.01, η^2^ = .61; Old/New: F(1, 14) = 15.90, p<.01, η^2^ = .53; New/New: F(1, 14) = 9.07, p<.01, η^2^ = .39) except the New/Old condition (p = .44), suggesting a general strategy of decreasing number of object fixations regardless of conditions.

To summarize the object fixation analysis, US participants looked longer at objects than did Singapore participants and they particularly looked more often at objects when the objects changed. The Singapore participants showed an invariant attentional bias regardless of the changing components of the picture.

### Background Fixation Data

**Figure 3:**
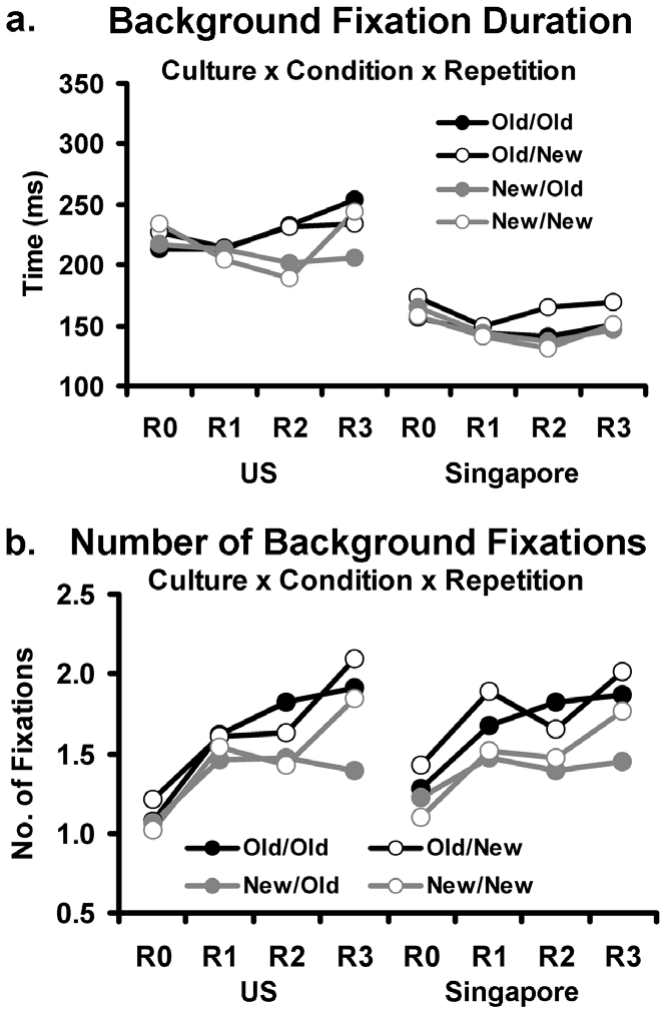
Mean background fixation data for US and Singapore participants. Mean background fixation durations (a) and number of background fixations (b) are shown across all quartet conditions (Old and New objects/backgrounds) and picture repetitions (R0 to R3).

An analysis of duration of background fixations yielded evidence for a main effect of culture, due to longer fixation durations to backgrounds in the US participants compared to the Singapore participants (F(1, 29) = 7.67, p<.01, η^2^ = .21; 220.6 ms vs. 151.6 ms). Culture was not involved in any higher order interactions. There was a significant main effect of repetition (F(3, 87) = 4.33, p<.01, η^2^ = .13) and an interaction of condition with repetition (F(9, 261) = 1.96, p<.05, η^2^ = .06; Figure 3a). Since there were no interactions with culture, we analyzed the linear trends of repetition across conditions using data collapsed across cultures. The analysis indicated a decrease in background fixation durations for the New/Old condition (F(1, 30) = 3.98, p = .06, η^2^ = .12) with no linear trends in the other conditions. The decrease in the New/Old condition was small and occurred because subjects had shorter fixations over time to backgrounds when objects were changing within repeated backgrounds.

The analysis of number of fixations to backgrounds yielded no significant effects of culture but main effects of condition (F(3, 87) = 7.84, p<.01, η^2^ = .21) and repetition (F(3, 87) = 70.27, p<.01, η^2^ = .71) and a repetition interaction with condition (F(9, 261) = 3.98, p<.01, η^2^ = .12), as shown in Figure 3b. Again, since there were no interaction effects of culture, further analysis of this interaction collapsed the data for the two cultures. Generally, subjects increased number of backgrounds fixations over time, except when the object changed against a stable background (New/Old vs. the mean of three conditions: Old/Old, Old/New, and New/New; F(1, 30) = 23.50, p<.01, η^2^ = .44), during which it was probably useful to make more object fixations.

Overall the background data yielded evidence for longer fixations to backgrounds in the US participants compared to the Singapore participants and evidence that attention to background was most likely when the objects were held constant. Note that although we would expect more fixations in the Singapore participants given their shorter fixation durations, there was no significant cultural difference in the number of fixations made within the fixed presentation duration. This may be because, in our methodology, eye gaze points that were part of a saccade were not counted as fixations and was the motivation for the following analysis utilizing the raw gaze datapoints rather than fixations.

### Gaze Distance and Proportion of Gaze Saccades between Objects and Backgrounds

The analysis of the eye fixation data showed that the US participants in general made longer fixations to both objects and backgrounds compared to shorter fixations in the Singapore participants. This interesting finding suggests that the Singapore participants may be visually examining a greater expanse of the pictures as well as making more saccades from objects to backgrounds (and back again) compared to the US participants. Such findings would be congruent with the notion that East Asians are frequently scanning the entire scene in order to develop a holistic representation. In order to test this hypothesis, we additionally obtained, for each picture, the total distance covered by raw eye-gaze movements (unprocessed for fixations) from one gaze datapoint to another, and the proportion of consecutive eye-gaze movements that were between objects and backgrounds, within objects, and within backgrounds, for every subject. These measures provide important indices of eye-movements in a way that is not limited by the fixation methodology above, which may fail to capture differences at the sub-fixation level.

**Figure 4:**
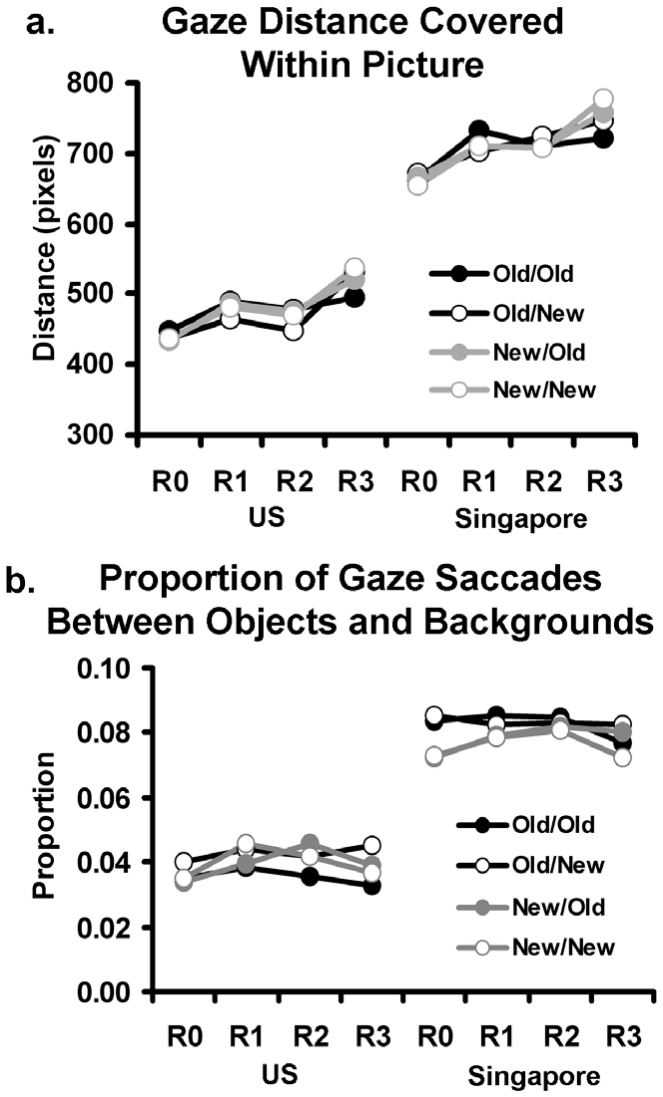
Eye gaze data for US and Singapore participants. Mean gaze distance covered within pictures (a) and proportion of gaze saccades between objects and backgrounds (b) are shown across all quartet conditions and picture repetitions.

Examination of the gaze distance data, measured in pixels, showed that the Singapore participants' eye-movements spanned greater distances within each picture compared to the US participants (Figure 4a). The same three way ANOVA as previously described performed on the gaze distance coverage revealed main effects of repetition (F(3, 87) = 45.65, p<.01, η^2^ = .61) and an interaction between repetition and condition (F(9, 261) = 2.82, p<.01, η^2^ = .09). Critically, this analysis also revealed a main effect of culture (F(1, 29) = 21.81, p<.01, η^2^ = .43) that did not interact with repetition or condition, and was qualified by Singapore participants having greater gaze distances (mean (s.d.) = 710.2 (35.9) pixels) than US participants (mean (s.d.) = 477.2 (34.7) pixels), supporting the idea that Singapore participants had greater gaze coverage of the stimuli and rarely settled onto one locus for long periods of time.

The same three-way repeated-measures ANOVA was next performed on the proportion of eye-gaze movements between objects and backgrounds. This analysis revealed a main effect of culture (F(1, 29) = 21.43, p<.01, η^2^ = .43) that did not significantly interact with condition or repetition (Figure 4b). The main effect occurred because the Singapore participants had a greater proportion of eye-gaze movements between objects and backgrounds (mean (s.d.) = 8.0 (.6) %) compared to the US participants (mean (s.d.) = 3.9 (.6) %). A second three-way ANOVA was performed on the eye-gaze movement within objects. This again revealed a main effect of culture (F(1, 29) = 4.68, p<.05, η^2^ = .14), that did not interact with condition or repetition, with the US participants having a greater proportion of eye-gaze movements within objects (mean (s.d.) = 69.1 (.3) %) than the Singapore participants (mean (s.d.) = 59.5 (.3) %). In a third ANOVA of eye gaze movements within backgrounds, there were no significant culture effects or interactions with culture. Overall these results are consistent with the notion of contextual processing in East Asians biasing them to process visual associations between the objects and backgrounds more than Westerners, and object-oriented processing in Westerners biasing them to process visual attributes of objects more than East Asians. Importantly, these cultural differences did not interact with condition or repetition, indicating their stability in the presence of data-driven changes.

## Discussion

Our findings suggest that there are stable cultural differences in the way East Asians and Westerners view pictures even when the external stimuli may capture attention in culturally non-preferred ways. The US participants had longer overall longer fixation durations to objects and backgrounds compared to the Singapore participants, a finding also seen in other studies [10], [16]. Further, when the US participants reacted to data-driven manipulations, it was largely in response to object rather than background manipulations. This is consistent with our hypothesis that object-focused processing in Westerners would result in attention being affected by novelty in object stimuli. We also showed that the Singapore participants were less reactive to the data-driven manipulations than the US participants, consistent with our hypothesis that holistic processing in East Asians would be associated with an equal distribution of attention to the scene, regardless of selective repetition of the respective picture components. It is important to note that although all participants modulated their eye-movements in response to stimulus novelty, the main effect of culture on fixation durations, gaze distances and eye-gaze movements between objects and backgrounds was much larger and stable relative to the effect of condition (the variable which manipulated stimulus change). Interestingly, modulation of fixation duration and the number of fixations for both cultures seemed largely in response to the novelty or repetition of objects, and only moderately sensitive to the manipulation of backgrounds, suggesting that object processing was important in both cultures, but less so for East Asians.

Critically, we demonstrated that the Singapore participants had greater eye gaze distance coverage within each picture and a greater proportion of their eye-movements alternating between viewing objects and scenes, whereas the US participants had less coverage and a greater proportion of their eye-movements remaining within the objects. These findings provide confirmation that East Asians are indeed attending to relationships between objects and backgrounds more than Westerners even at the sub-fixation level. In support of this, Jenkins et al. [34] provide recent neuroimaging evidence that demonstrates that East Asians are more affected than Westerners by incongruity of semantic relationships between objects and scenes, consistent with previous behavioral studies on the emphasis of functional relational categorizations in East Asians [35]. In that study, East Asians showed greater neural adaptation than Westerners to incongruent pictures in the lateral occipital regions, suggestive of greater attentional processing devoted to objects when the scene was incongruent. These findings point to a further necessary clarification that greater focus on contexts in East Asians is related to attention to both objects and backgrounds, rather than a disproportionate attention towards backgrounds only.

The findings of a stable cultural difference in measures of fixation duration, gaze distance and eye-movements between objects and backgrounds, with more dynamic effects observed for measures of the number of fixations suggest that these are separate aspects of visual attention processing that are modulated differently by culture. At the very least, the relationship between culture, attention and eye-movements are more complex than previously thought, and the results from this study serve as a platform for more detailed inquiry in future studies. Taken together, these eye-movement patterns, characteristic of context-focused and object-focused visual processing in East Asians and Westerners, respectively, constitute more specific findings regarding the nature of cultural differences in visual processing of objects and backgrounds.

The distinction between object- and context-focus in Westerners and East Asians has been conceptualized as generally greater attention to specific elements within a given picture. The findings in this study revealed more specific characteristics of this difference in visual biases between the two cultures. Object-focused processing in the US participants involved longer fixations durations, which would result in the identification of fewer visual loci of interest within a given viewing time, but more elaborative and detailed feature information obtained from each locus. This notion was also supported by our finding of more within-object gaze movements in the US compared to the Singapore participants. Further, object-focused processing in the US participants was related to a greater sensitivity to whether the central object in the picture was repeated or novel, emphasizing the importance of attributes associated with each object encountered rather than the different contexts of occurrence. Note that this does not mean that the US participants were completely unaffected by context changes since previous studies using Western participants have shown that behavioral responses and eye-movements to objects are modulated by contextual information [20], [23], [26], [29]. Additionally, in our own data, there is some evidence that the US participants' eye-movements were somewhat modulated by contextual differences in that the number of object fixations remained constant when backgrounds were changing (during the Old/New and New/New conditions) compared to when they were not, and was also greater for the New/Old compared to the New/New conditions during the last repetition (F(1, 15) = 15.46, p<.01, η^2^ = .51). Nevertheless, the clearest effects we observed in our data were primarily related to object changes, with background changes being more secondary, evolving during the fourth repetition. In contrast to the US participants, context-oriented visual processing in the Singapore participants involved shorter fixation duration, and a relative independence of eye-movements with respect to novelty or repetition of objects and backgrounds. The shorter fixation durations suggests that less detail may be acquired at each visual locus, but a greater number of critical samples of the picture can be identified. This mode of viewing pictures results from an emphasis on the whole picture as a holistic item, whereby the novelty of central objects and detailed object features are not as important as how each object relates to its context. This is supported by our finding of a greater proportion of eye-movements between objects and backgrounds in the Singapore compared to the US participants. While these projections from our findings seem consistent with respect to the existent literature on culture [3], [5], they are still speculations that should be verified in studies of even more specific mechanisms of cultural differences in visual processing and the impact on memory for visual details.

Like Chua et al. [10], we showed that there were longer fixation durations for the US compared to the Singapore participants for both object and background fixations. Unlike their study, we did not find a clear cultural difference in the number of fixations to backgrounds. This difference in results between the two studies may be related to some differences in experimental methodology. First, because we were interested in repetition effects, we utilized shorter stimulus duration in this study (1500 ms) compared to the Chua et al. [10] study (3000 ms). Because our stimulus durations were short, there may have been insufficient time for differences in number of fixations as a function of culture to emerge. Given that the Singapore participants had shorter fixation durations than the US participants in our study, it would be consistent to expect that if we used longer stimulus durations as Chua et al. [10] did, the total number of fixations in the Singapore participants in our study would eventually be greater than in the US participants. Second, there may have been an attentional difference in the way participants approached this rapid passive viewing paradigm in which the stimuli were presented on screen in quick succession, compared to the longer viewing time in Chua et al. [10] with constant objects and scenes. In the present study, subjects may have adopted more minimal sampling strategies due to an awareness of the limited time and the changing elements, rather than more elaborative encoding of the visual input [36], [37]. Third, the differences in task sets between Chua et al. [10] (pleasantness rating) and this present study (passive viewing) may interact with the cultural differences in visual attention resulting in the differences between our findings. Indeed, Shomstein et al.'s [38] study showed how scanning strategies can change when the task manipulates attentional priorities to overall spatial locations or more local object features. This one discrepancy between Chua et al.'s [10] data and ours, however, certainly does not detract from the other cultural effects on visual attention we found that replicate their data and are consistent with expectations given the known cultural biases observed across many other previous studies [9].

Interestingly, culture eye-tracking studies that used face stimuli demonstrated a different pattern of cultural differences than those reported in this present study, which used complex scenes. When looking at faces, it was the Western Caucasians in their sample that seem to scan more regions of the face, including the eyes, nose and lips, whereas East Asians tended to focus on either the central or eye regions [39], [40]. This result stands in contrast to our findings of greater scene scanning in Singapore participants with more focal eye-movements in US participants and suggests that the type of stimuli determines the culturally preferred visual processing strategy as well. Blais et al. [39] attributed the cultural differences when looking at faces to differences in social mores of interpersonal interaction in Western Caucasians and East Asians (e.g. East Asians consider it inappropriate to look directly at another person's eyes). Their findings are also consistent with the notion that analytic processing in Westerners may involve directing attention more to face features, whereas holistic processing in East Asians may emphasize the treatment of faces as a whole. Further experimental replication with different samples and paradigms are required to understand the interactions between cultural biases, types of stimuli, and task demands more specifically.

Unlike past studies of cultural differences involving visual processing, our study involved passive viewing rather than directed top-down processing (e.g., “memorize this picture”, “make a judgment about this picture”, etc). We submit that the passive viewing procedure is most similar to what happens in the real world, allowing subjects to engage in unconstrained scene analysis. For example, we often are placed in an environment with specific objects within either changed or removed, such as when a waiter removes our dishes in a restaurant. Likewise, we often encounter a constant object transitioning through various environments, such as when we bring a bag along with us from home to work. The fact that cultural differences still emerged in such an unconstrained situation adds confidence to the generalizability of culture effects on scene analysis. Moreover, although the passive viewing paradigm allowed participants to adopt idiosyncratic processes, both groups were given the same instructions. Thus, the most likely source of observed differences should be due to group differences – i.e. cultural experience.

In summary, there are cultural biases that operate in a top-down manner on the way individuals view objects and backgrounds in pictures. The bias appears to be that Westerners have longer fixation durations than East Asians, are relatively more affected by salient visual information about objects that capture attention in a data-driven manner, and have more within-object eye-movements possibly to acquire more detailed information about objects' attributes. In contrast, East Asian eye-movements are characterized by shorter fixation durations, are less affected by such focal visual changes, and have more eye-movements between objects and backgrounds possibly reflecting binding of objects to their contextual backgrounds. Finally, the different measures of eye-movements that characterize the number of fixations to objects and backgrounds, fixation durations, and eye-movements between objects and backgrounds may each reflect different aspects cultural biases on visual attention. Further work may involve whether these cultural visual biases can be changed through training or exposure to different environments [2].
